# Images in Primary Care Medicine: Point-of-Care Ultrasound in Gout

**DOI:** 10.7759/cureus.15096

**Published:** 2021-05-18

**Authors:** Dennis Espejo, Elizabeth Dearing, Kathleen Y Ogle, Maria Portela, Keith S Boniface

**Affiliations:** 1 Family Medicine, George Washington University School of Medicine and Health Sciences, Washington, DC, USA; 2 Emergency Medicine, George Washington University School of Medicine and Health Sciences, Washington, DC, USA

**Keywords:** point-of-care-ultrasound, gout crystals, primary care medicine, ultrasound-guided, crystal arthropathy

## Abstract

Gout is the most common crystal arthropathy and is frequently diagnosed and managed by primary care physicians. Point-of-care ultrasound (POCUS) is a valuable tool to aid in the diagnosis of gout via the identification of the double contour sign, aggregates of crystals, tophi, and erosions. In addition, POCUS can aid in the management of gout by recognizing early signs of gout, monitoring the effectiveness of urate-lowering therapy, and guiding aspiration and corticosteroid injection.

## Introduction and background

Crystal arthropathies are one of the most common causes of inflammatory arthritis worldwide [1]. Most crystal deposits are asymptomatic and can represent incidental findings on imaging, however, these accumulations of crystals can result in significant joint pain, swelling, subcutaneous nodules, limitation of joint function, and joint destruction. The most common type of crystal arthropathy is gout, caused by monosodium urate (MSU), which is characterized by crystal deposition in joints [2].

**Figure 1:**
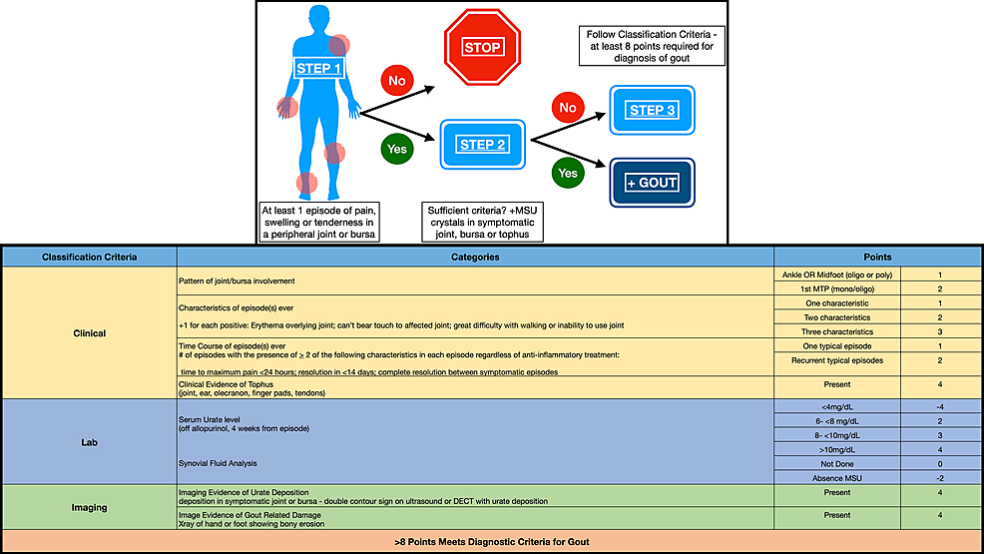
American College of Rheumatology and European League Against Rheumatism criteria for diagnosis of gout Step 1: Entry criterion necessary for the diagnosis - at least one episode of swelling, pain, or tenderness in a peripheral joint/bursa; if NO, the diagnosis of gout has been ruled out Step 2: Sufficient criteria (if YES, the diagnosis of gout is positive) - the presence of MSU crystals in a symptomatic joint, bursa, or tophus; if present, no need to proceed to Step 3 Step 3: Classification criteria - to be used if Step 2 sufficient criteria not met (at least 8 points required for the diagnosis of gout)

Gout is a common condition diagnosed and treated by primary care physicians. Between 2002 and 2008, gout accounted for approximately 7-million ambulatory visits at a cost of approximately 1-billion dollars [3]. In 2015 alone, 3.9% of the United States population, or over 8-million people, was afflicted with gout. While gout classically involves the first metatarsophalangeal joint, it can also affect the ankles, knees, midtarsal joints, fingers, wrists, and elbows [4]. Risk factors for the development of gout include age, family history, genetics, diet, and sex [5]. To facilitate the clinical diagnosis of gout, the American College of Rheumatology (ACR) and the European League Against Rheumatism (EULAR) developed a consensus set of guidelines in 2015 [6]. These guidelines provided steps and criteria to aid in the diagnosis of gout and included imaging criteria (Figure 1).

The use of POCUS in family medicine has been increasing because it improves clinical outcomes, reduces costs, improves the accuracy and efficacy of procedures, and improves patient satisfaction, especially in resource-limited areas [7-9]. Advances in technology and availability of equipment, as well as decreased size of ultrasound units, have fueled its adoption by clinicians. In addition, POCUS can provide real-time information at the bedside and avoids ionizing radiation. Due to this potential, the American Association of Family Physicians (AAFP) has published a POCUS curriculum for family medicine residencies that describes the scope of practice, competencies, and skills of POCUS for the family physician [10].

Ultrasound has been utilized by various medical specialties to evaluate musculoskeletal and rheumatological diseases. The objective of this paper is to describe the utility of POCUS in facilitating the diagnosis, treatment, and delivery of care for patients with gout and to demonstrate the value of POCUS to primary care practitioners when encountering patients with arthropathies.

## Review

Imaging in gout

The gold standard for the diagnosis of gout is the detection of negatively birefringent monosodium urate (MSU) crystals from synovial fluid or soft tissue/tophus aspirate. However, synovial aspiration may be deferred or unsuccessful for a variety of reasons, including patient intolerance of the procedure, scant fluid in the joint space or inability to aspirate fluid, and, depending on the clinical setting, lack of polarizing microscopy equipment. In addition, the synovial fluid analysis may not reveal MSU crystals in a significant (up to 25%) portion of patients with acute gout [11]. For these reasons, the diagnosis of gout is often made by a combination of laboratory and radiographic findings. Conventional radiography has traditionally been the primary imaging modality in the diagnosis and management of gout, but the use of other modalities has increased as technology has improved and become more readily available.

Plain radiography has been used for over a century in assessing gout and remains the most appropriate primary imaging choice for gout according to the American College of Radiology (ACR) [12-13]. Radiographs during an acute flare may reveal nonspecific findings such as soft tissue swelling and joint effusion. Joint spaces are maintained until later in the disease course [14]. The characteristic finding of gout on radiographs is an intraarticular or juxta-articular bone erosion, or cortical discontinuity, with overhanging and sclerotic margins [15]. However, bony erosions may be difficult to view due to the two-dimensional image, especially in areas of osseous overlap. Erosions are also a later finding of bony destruction from chronic inflammatory changes and may not be present until many years after the onset of gout. Because of these factors, the sensitivity for gout using plain radiography is quite low. Specificity, on the other hand, is very high and makes radiographs reasonably accurate [13]. Small tophi may be radiographically occult. If tophi are seen, they generally appear as an area of increased opacity in a round, ill-defined shape [12].

Computed tomography (CT) provides more detailed images than plain radiography when evaluating for crystal arthropathies. Crystals may be seen as hyperdensities in extra-articular and intraarticular areas compared to surrounding soft tissues [16]. CT can also show bony erosions and can evaluate areas of bone that are difficult to visualize with radiography. However, CT without contrast does not adequately evaluate soft tissues and, therefore, may not be useful in early gout. The main limitation of its regular use for gout is radiation exposure. CT with the addition of intravenous contrast can also provide information regarding inflammation but exposes the patient to contrast dye in addition to radiation and the findings remain rather nonspecific.

Dual-energy computed tomography (DECT) is growing in use and allows the differentiation of deposits in the tissue. DECT is created through X-ray absorption at different energy levels based on the material’s atomic number and allows for differentiation between calcium and MSU crystals. By being able to differentiate MSU from other substances, areas of urate deposition can be easily identified. A meta-analysis looking at the diagnostic performance of DECT in gout showed a pooled sensitivity of 84% and a specificity of 84%. False negatives are most frequent in patients with recent onset of disease and in cases of small deposits or non-tophaceous gout [17]. The utility of DECT may be limited by the availability of equipment, cost, and exposure to radiation, making monitoring of disease using this modality less desirable. Still, DECT is noninvasive and can aid in the diagnosis of patients with more complicated presentations such as spinal gout [18]. In a study comparing DECT to ultrasound in the diagnosis of gout in upper and lower extremity joints, the authors found that DECT was superior to US in the upper limb joints but showed no difference in the lower limbs [19]. Other studies have found similar results that US performs as well as DECT in the lower extremities and has poorer performance than DECT in the upper extremities [20-21]. Although all studies had a small number of patients, it may be reasonable to conclude that US would be a useful screening tool but DECT may still need to be performed in the upper extremities if US results are negative and clinical suspicion still exists. Still, the ACR/EULAR classification criteria list either the double contour sign on ultrasound or areas of MSU deposition on DECT as equally acceptable [6].

MRI is another option for the evaluation of arthropathies due to its excellent visualization of soft tissue and synovial inflammation related to gout. MRI is also able to visualize soft tissue tophi and bony erosions. However, many of the typical findings are non-specific inflammatory changes in soft tissues and bones, which can also be seen in infections and tumors. Other imaging modalities or tests are often performed to differentiate the etiology of the inflammation and confirm the diagnosis [16]. MRI has the benefit of not exposing the patients to ionizing radiation; however, the cost of MRI can be prohibitive.

Overall, each of these imaging choices has its advantages and disadvantages with regards to the evaluation of gouty arthritis. While the ACR recommends radiographs for initial evaluation of suspected gout, the EULAR recommends the use of ultrasound and DECT due to their high specificity. MRI and CT can provide some information including inflammatory changes as well as crystal deposits, but the findings are less specific for gout than DECT and US.

Ultrasound features of gout

There are many practical advantages in the use of ultrasound for the evaluation of gout compared to the previously mentioned imaging modalities. These include ultrasound’s ready availability at the bedside, low operational costs and time, real-time information for clinicians, and the ability to perform a rapid examination of multiple locations [22-24]. The increase of skilled clinician sonographers and of ultrasound equipment makes POCUS an ideal bedside tool for the assessment of the patient with crystal arthropathy. Ultrasound of patients with suspected gout focuses on the identification of crystal deposition in the joints and soft tissues through several characteristic sonographic findings. These findings include the double contour sign, aggregates or clusters of crystals, tophi, and erosions [25].

**Figure 2:**
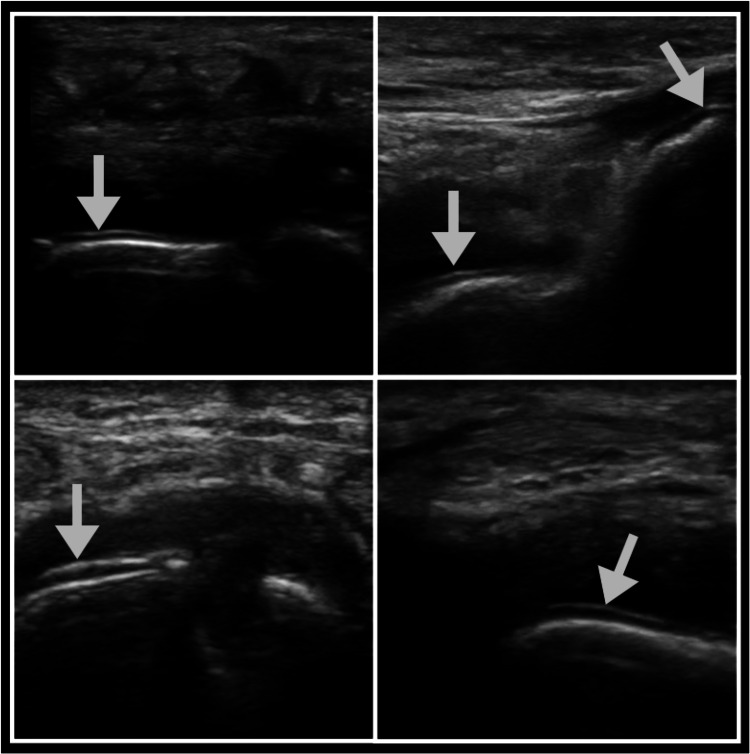
Double contour sign The arrows highlight the double contour sign.

When MSU crystals precipitate and are deposited along the hyaline cartilage of the articular surface, a hyperechoic bright band that parallels the hyperechoic bony cortex is visualized and called the double contour (DC) sign. The DC sign may be either regular or irregular and may be continuous or intermittent [25]. A false-positive may result from artifactual reflections from the cartilaginous surface; however, this artifact disappears with angulation of the probe while the DC sign will be visualized independently of the insonation angle [6]. The double contour sign has been shown in studies to have high specificity and moderate sensitivity for the diagnosis of gout. Two recent meta-analyses had similar pooled sensitivities of 65.1% and 66% and specificities of 89% and 92% [26-27]. Additionally, the inter-observer agreement for the double contour sign is excellent (0.98) [25]. Due to its high specificity, the DC sign is included in the ACR/EULAR gout classification criteria; the only ultrasound finding included in the criteria [6,27]. The only moderate sensitivity is likely due to difficulty visualization and poor acoustic windows at small joints such as those of the hands (Figure 2). See narrated video for features of the double contour sign (Video 1).

**Video 1:** Double contour sign

**Figure 3:**
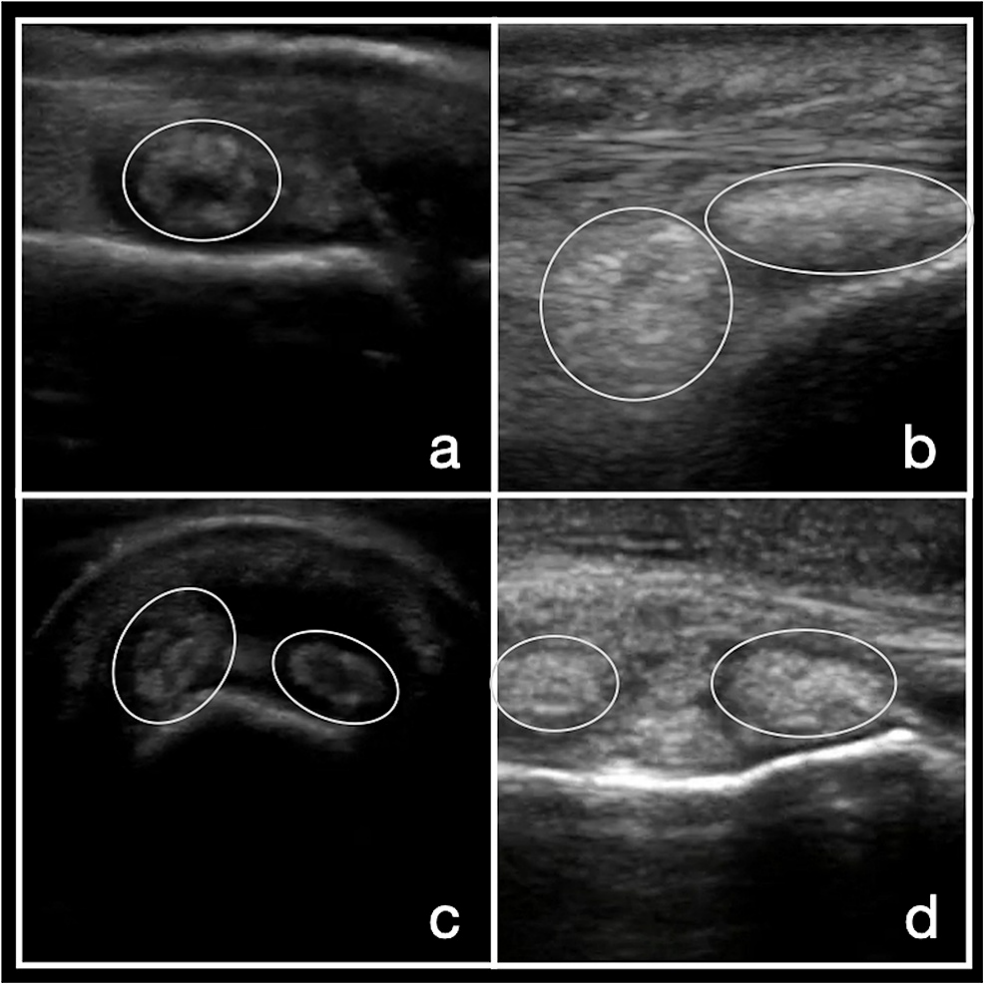
Tophi Images a-d demonstrate tophi in the synovium of multiple joints.

Tophi are heterogeneous hyperechoic lesions with poorly defined margins with or without areas of acoustic shadowing and circumscribed by a hypoechoic rim. Tophi contain MSU crystals and can be found in synovium, tendons, and soft tissues, including the fingers, overlying the olecranon, the great toe, and the pinna of the ear. The presence of tophi has a sensitivity of 56% and a specificity of 94% for the diagnosis of gout [26]. Inter-observer agreement for the identification of tophi is 0.71 (Figure 3) [25]. See narrated video for features of tophi (Video 2).

**Video 2:** Tophi

**Figure 4:**
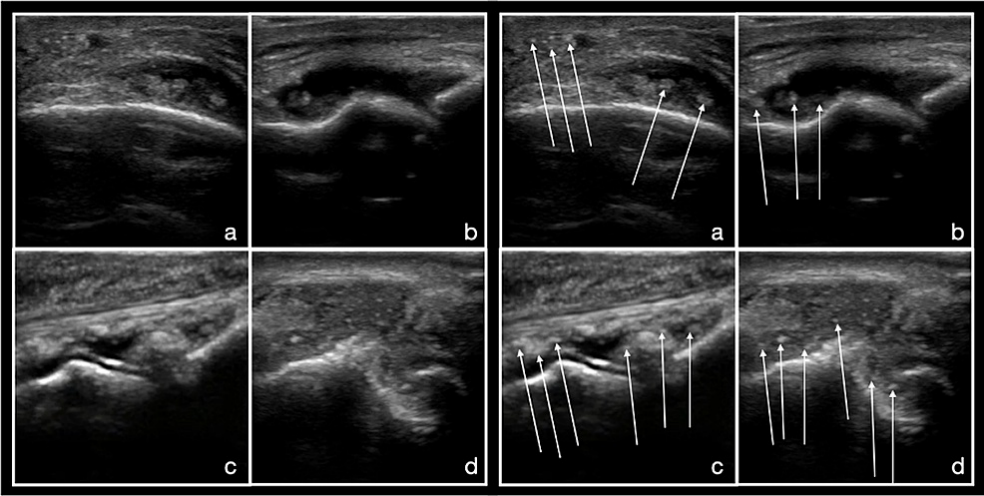
Aggregates On the left, a-d are views of the joint of interest without labels. The images on the right are the same images with arrows highlighting several highly reflective aggregates within the joint space and soft tissue.

Aggregates have also been defined in the literature and are believed to be deposits of crystals in the soft tissues, which are not large enough to be defined as a tophus. These aggregates have been described as heterogeneous hyperechoic foci with high reflectivity that does not change based on the angle of probe or gain [25]. Aggregates seem to be less well-defined, as the inter-observer agreement is only fair, although this may be related to their low prevalence [28]. Given these factors, they appear to be less useful than tophi in the diagnosis of gout (Figure 4). See narrated video for features of aggregates (Video 3).

**Video 3:** Aggregates

MSU crystals may appear in the joint fluid or in effusions. These crystals are mobile and echogenic, creating a “starry sky” appearance. Compression of the joint fluid with the ultrasound probe followed by a release of the pressure can cause movement of the fluid and crystals creating a “snowstorm” appearance from turbulent flow [14]. A recent meta-analysis showed a pooled sensitivity of 30.8% and a specificity of 90.6%. This is the least sensitive finding compared to the DC sign, tophi, and erosions; however, it is very highly specific. 

Erosions are defined as intra- and/or extra-articular discontinuity of the bone surface in two perpendicular planes [25]. These lesions are usually rounded or oval-shaped in gout and have a sclerotic rim causing a “punched-out” appearance and frequently with overhanging edges. Bony erosions, as well as the overhanging edges, are caused by the expansion of the tophus into the bony with new bone formation. The most frequently involved site for gout is the first metatarsophalangeal joint. Erosions are more common with increased age, chronic gout, increased number of tophi, and synovial hypertrophy [29]. See video for features of erosions (Video 4).

**Video 4:** Erosions

While each of these findings (double contour sign, tophi, snowstorm, and erosions) have good specificity for the diagnosis of gout, they may carry more diagnostic power if visualized together. Nonspecific findings of joint inflammation can also be seen in patients with an acute gout flare. These are secondary to inflammatory changes and include joint effusion, synovitis, and increased vascularity with Doppler in synovial tissue and in tophi. See narrated video for features of hypervascularity (Video 5).

**Video 5:** Hypervascularity

Integration of ultrasound into the management of gout

While synovial fluid or soft tissue analysis for MSU crystals is the gold standard for diagnosing gout, this procedure is, at times, difficult to perform, especially for small joints, is unsuccessful, or is not able to be performed due to lack of available polarizing light microscopy. Therefore, diagnostic criteria have been developed to aid in the diagnosis of gout (Figure 1) [6]. A significant portion of the diagnoses of gout is based upon clinical presentation and treated based on a presumptive diagnosis of gout. In addition to joint pain, chronic gout has been associated with adverse cardiovascular and renal outcomes which necessitate the importance of diagnosing and treating gout early in the disease course [2,30-34].

In addition to the identification of MSU crystal deposition by DECT and imaging evidence of gout-related joint damage by plain radiographs, the double contour sign by ultrasound is included in the ACR/EULAR consensus guidelines for gout classification criteria [6]. Furthermore, the EULAR recommends the use of ultrasound for patients with suspected gout flare or chronic gouty arthritis since ultrasound can detect tophi not evident on clinical examination or the DC sign, which is highly specific for gout. Furthermore, the DC sign can be found during acute flares as well as during asymptomatic periods of MSU crystal deposition. For these reasons, as well as the benefits, ultrasound has been prioritized over other imaging modalities for the diagnosis of gout [18]. Ultrasound can also play a role in the management of gout long-term with the benefits of low cost and no additive radiation with recurrent evaluation.

Recognizing early signs of gout

Patients with hyperuricemia can be asymptomatic. However, ultrasound images of these asymptomatic individuals with hyperuricemia can be demonstrated to have ultrasonographic signs of gout. Stewart et al. conducted a systematic review and meta-analysis to determine what signs and locations were frequently involved in asymptomatic patients. They found that the double contour sign in the first metatarsophalangeal joint (MTP1) and femoral condyle and tophi in the MTP1 were most prevalent [35]. This can lead to early recognition of subclinical gout and lead to measures like dietary modification and urate-lowering therapy to prevent disease progression.

Monitoring urate-lowering therapy

While hyperuricemia does not always result in the development of gout, lowering serum urate levels is the goal in gout treatment to reduce flares and complications. The goal of gout treatment is to reduce serum urate levels (SUL) to a normal level of <6 mg/dl [36]. This is evaluated by serial blood tests once SUL lowering treatment is initiated. Studies have explored the use of ultrasound to follow response to SUL lowering treatment. Multiple studies have shown a reduction or resolution of the DC sign once patients who were on SUL lowering drugs obtained a normal SUL of <6 mg/dl [37-38]. In the USEFUL (UltraSound Evaluation in Follow-up of Urate-Lowering therapy in gouty patients) study, the authors also showed a decrease in the size of tophi and a disappearance of the double contour sign on ultrasound after urate-lowering treatment [24]. Overall, recent studies have shown that ultrasound is a promising noninvasive way to evaluate response to serum urate-lowering treatment. Future studies should evaluate the impact of serial ultrasound on compliance with SUL lowering therapy.

Guiding aspiration

The gold standard for diagnosing gout is synovial fluid analysis. Often, this is a difficult procedure to perform or results in a “dry tap” due to associated pain, a difficult or small joint space to access, or not being in the right location for aspiration. Ultrasound has been shown to increase the success rate of knee arthrocentesis, increase the amount of fluid aspirated, and decrease procedure pain by 50% (Video 6) [39].

**Video 6:** Arthrocentesis

Although there is clinical controversy on whether ultrasound should be used to guide every joint aspiration, there is general agreement that ultrasound can guide aspiration of smaller joints and smaller effusions that would otherwise be unable to be aspirated using external landmarks alone [40-41].

Guiding steroid injection

The first-line treatment of acute gout includes nonsteroidal anti-inflammatory drugs (NSAIDs), colchicine, and oral steroids [4,36,42]. However, for patients with renal disease in whom the use of NSAIDs and colchicine is limited or those with diabetes where systemic steroids can lead to uncontrolled hyperglycemia, an alternative treatment is intraarticular corticosteroid injections. Similar to ultrasound-guided joint aspiration, ultrasound-guided joint injections have been shown to be safer and more accurate than a landmark-based approach. The most common site for an acute gout attack is the MTP1, a challenging joint to access with arthrocentesis by landmark [4]. In a small study, Kang et al. evaluated the safety and efficacy of intraarticular corticosteroid injections in the MTP1. The authors found ultrasound-guided intraarticular injection to be safe as evidenced by no adverse reactions. They also state that the procedure was effective, providing a significant reduction in pain at 24 and 48 hours; however, there was no control group for comparison [43]. Still, it seems safe to assume that direct visualization of the joint space with ultrasound would lead to improved outcomes as compared to injection of a swollen joint with distortion of normal anatomy.

Limitations of POCUS for gout

While it is clear that ultrasound can play an important role in the diagnosis and management of gout, limitations do exist in its applicability to daily use in the clinical environment. POCUS use is growing, especially with more affordable and portable machines, including hand-held devices, but training and quality improvement programs are needed to ensure quality care is being provided. Significant foundations of the literature of ultrasound of patients with suspected gout have been written by radiologists and rheumatologists with expertise in soft tissue and musculoskeletal ultrasound. There is a need for research of POCUS-aided diagnosis and treatment in the clinic setting by the primary care clinicians who treat the majority of these patients. Still, we believe that with adequate training, POCUS use in the evaluation of inflammatory arthritis will become more widespread and an integral part of gout evaluation and management in the outpatient setting.

## Conclusions

Ultrasound is a powerful tool for trained clinicians in the outpatient setting for the evaluation of arthralgia and is part of the diagnostic criteria for the clinical diagnosis of gout. Clinicians are increasingly utilizing POCUS to inform clinical decisions and guide procedures at the bedside in real time. Gouty arthritis causes significant morbidity to patients, and these patients frequently present first to their primary care doctor. POCUS by family medicine physicians not only allows for more rapid identification and diagnosis of patients with gout but also aids in improving the sensitivity of arthrocentesis, the accuracy of intraarticular steroid administration, and the monitoring of response to therapy.
